# DNMT1 mutant ants develop normally but have disrupted oogenesis

**DOI:** 10.1038/s41467-023-37945-4

**Published:** 2023-04-18

**Authors:** Iryna Ivasyk, Leonora Olivos-Cisneros, Stephany Valdés-Rodríguez, Marie Droual, Hosung Jang, Robert J. Schmitz, Daniel J. C. Kronauer

**Affiliations:** 1 Laboratory of Social Evolution and Behavior, The Rockefeller University, New York, NY USA; 2 Howard Hughes Medical Institute, New York, NY USA; 3 Department of Genetics, University of Georgia, Athens, GA USA

**Keywords:** Evolutionary biology, Evolutionary developmental biology, Oogenesis, DNA methylation

## Abstract

Although DNA methylation is an important gene regulatory mechanism in mammals, its function in arthropods remains poorly understood. Studies in eusocial insects have argued for its role in caste development by regulating gene expression and splicing. However, such findings are not always consistent across studies, and have therefore remained controversial. Here we use CRISPR/Cas9 to mutate the maintenance DNA methyltransferase DNMT1 in the clonal raider ant, *Ooceraea biroi*. Mutants have greatly reduced DNA methylation, but no obvious developmental phenotypes, demonstrating that, unlike mammals, ants can undergo normal development without DNMT1 or DNA methylation. Additionally, we find no evidence of DNA methylation regulating caste development. However, mutants are sterile, whereas in wild-type ants, DNMT1 is localized to the ovaries and maternally provisioned into nascent oocytes. This supports the idea that DNMT1 plays a crucial but unknown role in the insect germline.

## Abstract (web summary)

The role of DNA methylation in insects is poorly understood. Here, the authors knock out the DNA methyltransferase DNMT1 in an ant and find no obvious effects on development, rather showing that this enzyme seems to play a crucial role during early oogenesis.

## Introduction

How epigenetic processes regulate development and behavior is a major area of research^1^. Among the most prominent epigenetic mechanisms is DNA methylation, a covalent modification to cytosine that gives rise to 5-methylcytosine. This modification is catalyzed by two types of enzymes, de novo DNA methyltransferases (DNMT3) and maintenance DNA methyltransferases (DNMT1). While DNMT3 primarily targets previously unmethylated cytosines, DNMT1 mostly copies DNA methylation during DNA replication^2^. In most organisms, the majority of cytosine methylation occurs in a CpG context, i.e., a cytosine base followed by a guanine^3^. However, the presence and amount of CpG methylation is highly variable across organisms^4,5^.

The function of DNA methylation has mostly been studied in mammals, where it is primarily targeted to cis-regulatory elements, transposons, and gene bodies^3,6^. Promoter and transposon methylation has important functions in gene regulation and usually acts to downregulate or silence expression, also in the context of genomic imprinting^7–10^. However, across the tree of life, DNA methylation can play different roles^11^. Honeybees and ants possess full complements of the DNA methylation machinery^12–15^, which has been met with considerable excitement for two main reasons^6,16–26^. First, the commonly used invertebrate genetic models yeast, *Caenorhabditis elegans*, and *Drosophila melanogaster*, lack parts of the machinery and, therefore, CpG methylation, opening the possibility that social insects could serve to better understand the role of DNA methylation in invertebrates, which remains poorly known^4^. Second, social insects show extreme forms of developmental and behavioral plasticity, and it has been argued that DNA methylation might play important roles in caste development^26–29^, the regulation of behavioral roles^6,29–31^, and the evolution of genomic imprinting as a result of social conflicts^32,33^.

Several studies have reported correlations between DNA methylation patterns and queen vs. worker castes^12,34,35^, while others have attempted experimental manipulations and reported effects on caste development or behavior either via changes in gene expression or alternative splicing^36–38^. While these studies have greatly contributed to our understanding of DNA methylation in social insects, others have failed to find consistent correlations between DNA methylation and reproductive castes, and some of the core functional results have not been replicated^13,29,39^. Also, DNA methylation in insects is preferentially found in the exons of constitutively expressed and evolutionarily conserved housekeeping genes, which seems to contradict the conjecture that it is associated with dynamic gene regulation^13,40–42^. Finally, the few functional studies of DNA methylation in social insects have been limited to pharmacological manipulations or RNAi knockdowns of DNA methyltransferases^36–38^, calling for additional studies that employ more definitive molecular genetics approaches.

Here, we study the role of DNA methylation in the clonal raider ant, *Ooceraea biroi*. Unlike honeybees and most ants, *O. biroi* does not have queens, and all workers in a colony reproduce asexually and clonally^43,44^. Therefore, mutant lines can be established from any individual with germline transmission of natural or experimentally induced mutations^45^. At the same time, there still is phenotypic plasticity along the worker-queen spectrum among workers of this species, with smaller “regular workers” with two ovarioles and no eyespots, and larger, more queen-like “intercastes” with four to six ovarioles and rudimentary eyespots^46,47^. Using CRISPR-mediated mutagenesis of *DNMT1*, we show that ants deficient in DNA methylation still develop normally and do not show obvious alterations to caste phenotypes. However, these ants are sterile, adding to recent studies in other species suggesting that DNMT1 plays an important yet poorly understood role during insect oogenesis^48–52^.

## Results and discussion

**Fig. 1:**
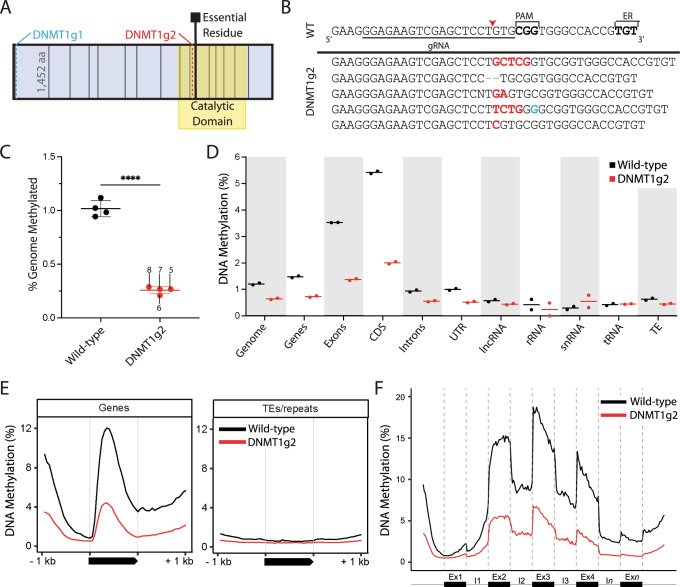
Mutations in the DNMT1 catalytic domain result in decreased DNA methylation. **A** Clonal raider ant DNMT1 protein model. Vertical solid lines indicate exon boundaries; broken lines indicate CRISPR/Cas9 target cut sites. The DNMT1g1 (blue) target site is early in the second exon, while the DNMT1g2 (red) target site is in the catalytic domain (yellow), upstream of an essential cysteine residue (black square). **B** Top: Wild-type (WT) *DNMT1* sequence at the DNMT1g2 target site, with the codon for the essential residue (ER) and the protospacer adjacent motif (PAM) in bold. Guide-RNA (gRNA) sequence underlined. Red arrowhead indicates predicted cut site. Bottom: Mutant alleles observed in G0 adults show insertions (red), deletions (gray) and base changes (blue). **C** DNMT1g2 mutants have decreased genome-wide methylation compared to wild-type ants reared in parallel, consistent with a functional defect in DNMT1 (two sided unpaired T-test: *p* < 0.0001; *n* = 4 animals per condition). Error bars show standard deviation around the mean. Each data point represents low coverage WGBS of DNA extracted from the remaining tissue of a single ant after brain and ovary dissection. Data point labels correspond to animals #5-8 in Supplementary Table 1. Methylation levels are corrected for bisulfite non-conversion. **D** Percentages of methylated cytosines for different genomic features based on high coverage WGBS of two biological replicates of wild types (black) and DNMT1g2 mutants (red) from (**C**) (bars denote means). **E** DNA methylation patterns in wild types (black) and DNMT1g2 mutants (red) for genes and TEs/repeats. The 1 kb upstream and downstream regions along with the gene body region (black bar) are displayed. **F** Gene body methylation profile for different positions of exons and introns across the *O. biroi* genome. Exons and introns are shown separately for wild types (black) and DNMT1g2 mutants (red). Source data are provided as a Source Data file.

DNMT1 copies DNA methylation patterns during DNA replication^2^, and enzyme loss-of-function should result in genome-wide loss of CpG methylation. The *O. biroi DNMT1* gene (NCBI GenBank, Gene ID: 105286975) is composed of 16 exons (Fig. 1A), and RNA sequencing (RNA-seq) data show that the short first exon is alternatively spliced (Supplementary Fig. 1). We therefore designed two CRISPR guide RNAs, one to induce frameshift mutations in the second exon (DNMT1g1), and one to target exon 11 of *DNMT1*, just upstream of a conserved residue in the catalytic domain that is essential for enzyme function in mice (DNMT1g2) (Fig. 1A)^53^.

### Early frameshifts in DNMT1 do not lead to loss of function

We first injected 5152 freshly laid eggs with Cas9 enzyme and the DNMT1g1 guide^45^, and recovered two unique stable mutant lines in which both alleles were frameshifted (Supplementary Fig. 1A). Unexpectedly, low-coverage whole-genome sequencing of bisulfite-treated DNA (WGBS) showed that DNA methylation levels in these mutants were indistinguishable from wild-type ants (Supplementary Fig. 1B), implying that the DNA methylation function of DNMT1 was not affected. Both mutant strains showed no obvious phenotypic abnormalities, produced viable eggs at normal rates (Supplementary Fig. 1C) and had normal lifespans (Supplementary Fig. 1D). Whole-body RNA-seq of individuals from one of the two lines (−7bp/−8bp) and matched wild-type controls found no differences in *DNMT1* expression. Additionally, even though the genomic frameshift mutations were reflected in RNA-seq reads, we did not observe any major alternative splicing events in mutated *DNMT1* (Supplementary Fig. 1F). Taken together, this suggests that gene function can be rescued when *DNMT1* is frameshifted in early exons. Even though we do not know the exact rescue mechanism in this case, gene rescue is a known problem in functional genetic studies, and can occur in a number of ways, including translation reinitiation or undetected alternative splicing/exon skipping^54,55^. These mutants nevertheless provide a useful control in the context of the current study, because they demonstrate that we can mutate the *DNMT1* gene with CRISPR/Cas9 reagents without causing adverse fitness effects or other non-specific phenotypes if the mutation does not measurably compromise the function of the DNMT1 enzyme.

### DNMT1 loss-of-function mutants have reduced DNA methylation

Across two experiments, we then injected 5643 young eggs with Cas9 enzyme and the DNMT1g2 guide to generate 33 G0 adults. Because G0 adults have not inherited mutations via the germline, they can be genetic mosaics. However, Sanger sequencing and subsequent analyses (see below and Supplementary Table 1) showed no evidence of wild-type alleles in seven G0 females and one G0 male, whereas one G0 female had both mutant and wild-type alleles. We were not able to obtain genotypes for three of the 33 G0s, but one of them was confirmed later as a loss-of-function mutant via immunohistochemistry and WGBS (see below). Because we could not establish stable lines of DNMT1g2 mutants (see below), this female and the seven females without detected wild-type alleles were used in all subsequent experiments. Individual details for these eight animals are given in Supplementary Table 1, and we confirmed several mutant alleles with small insertions and deletions at the target site via Sanger sequencing (Fig. 1B). The remaining G0 adults were wild-type females and served as matched experimental controls.

To assess the effect of these mutations on DNA methylation, we extracted DNA from whole bodies (minus ovaries and brains) of four mutants (Supplementary Table 1, individuals #5-8) and four wild-type controls and conducted low-coverage WGBS. The mutants had greatly reduced average genome-wide DNA methylation levels (0.26%; 95% CI: 0.21–0.31%) compared to the wild-types (1.02%; 95% CI: 0.90–1.24%) (Fig. 1C). We then conducted high-coverage WGBS for two of these mutants and two of the wild-types, achieving 14X coverage of the genome on average and greater than 99% sodium bisulfite conversion rates (Supplementary Table 2). The striking reduction in DNA methylation was constant across all genomic features, regardless of their location in relation to genes, repeats or transposons (Fig. 1D–F, Supplementary Fig. 2). As expected, DNMT1 loss of function thus results in a substantial reduction in DNA methylation. These data are consistent with the expected function of DNMT1. The remaining low methylation levels in DNMT1g2 mutants could be due to a number of possible mechanisms, including the enzymatic activity of DNMT3 or signal stemming from cells that did not divide after injection of Cas9. No wild-type reads were detected at the *DNMT1* target site in the G0 DNMT1g2 mutants with high-coverage WGBS data, providing additional evidence that mosaicism in G0s is low (Supplementary Table 3).

### DNMT1 loss-of-function mutants are not more queen-like

**Fig. 2:**
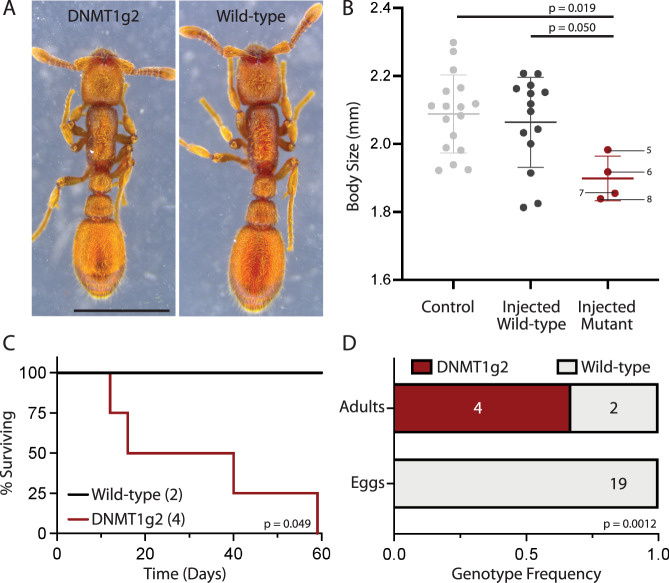
DNMT1 mutants develop normally, but have impaired survival and reproduction. **A** Images of DNMT1g2 mutant and wild-type animals. DNMT1g2 mutants complete development and are grossly indistinguishable from wild types in external anatomy despite lacking the functional DNMT1 enzyme (scale bar = 1 mm). **B** Mutants appear smaller in total body size (one-way ANOVA *p* = 0.0248; *n* = 4 mutant animals, 17 control animals, and 14 wild-type animals that were injected at the egg stage along with the mutants). Body size was calculated as the sum of the head, thorax, petiole, post-petiole and gaster lengths (Supplementary Fig. 3). Horizontal bars show pairwise comparisons using Tukey’s multiple comparisons test. In injected wild-type ants, CRISPR injections at the egg stage failed to produce mutations in *DNMT1*. Labels of individual data points correspond to animals #5-8 in Supplementary Table 1. **C** DNMT1g2 mutants show survival deficits relative to wild types (log-rank test: *p* = 0.049). Sample sizes are given in parentheses. **D** Eggs laid by all G0 adults were collected and sequenced, ratios of wild-type to mutant eggs compared to ratios of wild-type to G0 adults are shown. Numbers indicate sample sizes. All sequenced eggs were wild type, while only two of the six G0 adults carried wild-type alleles (two-sided Fisher’s exact test: *p* = 0.0012). Source data are provided as a Source Data file.

In mammals, DNA methylation is vital to multiple aspects of development, including genomic imprinting^56^. Accordingly, experiments that interfere with DNMT function result in DNA replication arrest^57,58^ and embryo lethality^59,60^. This contrasts with our findings in *O. biroi*, where both males and females (Fig. 2A) complete development despite DNMT1 loss-of-function and significantly reduced DNA methylation. This suggests that the roles of DNMT1 and DNA methylation during development differ fundamentally between mammals and insects. However, the effects of DNA methylation on insect development could be more subtle, and previous work on ants^36^ and honeybees^37^ suggested that reduced DNA methylation modulates caste development and results in larger adults. In contrast to these findings, morphometric analyses of DNMT1 loss-of-function mutants showed that their morphology is similar to wild-type ants that were reared under identical conditions, and that their overall body size is possibly smaller on average (Fig. 2B, Supplementary Fig. 3). This finding contradicts the notion that DNA methylation increases in response to a reduced diet or other environmental factors during development^27^ and then mediates molecular changes that result in minor worker rather than major worker development in ants^36^, or worker rather than queen development in honeybees^37^. However, we currently cannot rule out the alternative possibility that the role of DNMT1 and DNA methylation in caste development differs between the clonal raider ant, which lacks extreme caste polymorphism, and other eusocial Hymenoptera.

### DNMT1 loss-of-function mutants are sterile

To assess the effect of DNMT1 loss-of-function on survival and fertility, we monitored a cohort of eight G0 adults from eclosion onward. By 60 days, we had collected four of the G0s shortly after they had died, two had likely died but carcasses could not be found (the ants sometimes dismember dead nestmates), and two remained alive. Sanger sequencing of DNA from whole body extracts showed that the four individuals that had died were DNMT1 mutants with no evidence of wild-type alleles (Supplementary Table 1, #1-4), while the two surviving individuals were wild type with no evidence of mutant alleles. The survival of DNMT1 mutants was significantly decreased relative to wild-type controls (Fig. 2C). To put this finding into context, median survival of wild-type *O. biroi* under similar rearing conditions was 258 days, with a minimum survival of 106 days among 21 individuals (Supplementary Fig. 1D). This shows that loss-of-function mutations in *DNMT1* severely compromise longevity. During the 60 days of this experiment, we collected 19 G1 eggs produced by the G0 adults and genotyped them at the *DNMT1* target locus. All these eggs were wild type. This is in stark contrast to the genotypes of G0 adults (Fig. 2D, Supplementary Table 1 #1-4) and implies that DNMT1 loss-of-function mutations result in sterility. That sterility is caused by the injection of CRISPR/Cas9 reagents or DNMT1 mutations per se is unlikely, because G0 adults mutated with the DNMT1g1 guide RNA did not show this phenotype. This opens the possibility that the decreased lifespan of DNMT1 mutants is not a direct consequence of reduced DNA methylation levels, but rather a result of the compromised reproductive system, for example by disrupting endocrine functions.

**Fig. 3:**
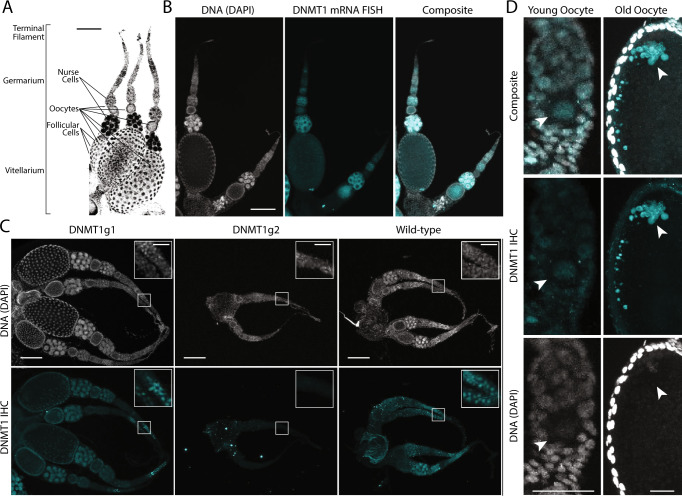
DNMT is maternally provisioned into the oocyte. **A** DAPI stain (black) of DNA showing the anatomy of a clonal raider ant ovary with three ovarioles. Oocytes arise from stem cells in the germarium and travel to the vitellarium as they develop. Oocytes are surrounded by supporting follicular cells and adjacent nurse cells (scale bar = 100 μm). **B** mRNA FISH of *DNMT1* in clonal raider ant ovaries. *DNMT1* staining is observed in nurse cells, germarium cells, and oocytes (scale bar = 100 μm). **C** Protein immunohistochemistry (IHC) of DNMT1 in the ovary of DNMT1 mutants (left, center) and a control wild-type ant (right). DNMT1 staining is visible in the germarium, nurse cells, and follicular cells of wild-type and DNMT1g1 mutant ovaries. No staining is apparent in the DNMT1g2 mutant, corresponding to individual #7 in Supplementary Table 1 (scale bars = 100 μm). Boxes show magnifications of the germaria (scale bars = 25 μm). Note that the wild-type ovary shown here happens to be inactive, while the DNMT1g1 mutant ovary is active, containing large oocytes. See (**B**) and Supplementary Fig. 4 for comparable images of active wild-type ovaries. **D** DNMT1 antibody staining in young (left) and old (right) oocytes. DNMT1 is present in young oocytes and appears limited to the nucleus (arrowhead). In old oocytes, DNMT1 localizes in distinct clusters in the periphery of the oocyte, with large amounts of protein co-localizing with DNA (arrowhead). Orientation of oocytes is the same as in (**A**) (scale bars = 25 μm).

To better understand the putative role of DNMT1 in reproduction, we characterized *DNMT1* mRNA and protein in the ant ovary. Each of the two ovaries of an *O. biroi* ant is composed of one to three ovarioles (two to six ovarioles per ant), which can be subdivided into a vitellarium, germarium and the terminal filament (Fig. 3A), similar to ovarioles of other insects^61^. In the ovarioles, each oocyte originates in the germarium, and travels to the vitellarium, where it is surrounded by follicular cells and an adjacent bundle of nurse cells (Fig. 3A). Nurse cells are derived from the same progenitor cell as the associated oocyte and are therefore of germline origin. Using mRNA fluorescence in situ hybridization (FISH), we detected *DNMT1* mRNA in the germarium, as well as the nurse cells and oocytes within the vitellarium (Fig. 3B, Supplementary Fig. 4). The broad expression of *DNMT1* in *O. biroi* ovaries is consistent with qPCR data from *Solenopsis invicta* fire ants, showing that *DNMT1* is expressed in ovaries^62^. We then stained ovaries from wild-type and mutant *O. biroi* using a commercial antibody against the conserved catalytic domain of mammalian DNMT1. Consistent with the mRNA FISH pattern, we observed DNMT1 protein within the nuclei of cells in the germarium, nurse cells, follicular cells and oocytes (Fig. 3C, Supplementary Fig. 4). Positive and specific staining was apparent in wild-type ants and DNMT1g1 mutants, but not in the ovaries of three DNMT1g2 mutants (Fig. 3C, Supplementary Fig. 5). The positive staining in DNMT1g1 mutants confirms that, despite the early frameshifts, they still produce some form of functional DNMT1 protein, highlighting the importance of independently assessing the effects of targeted mutagenesis on gene function.

The lack of staining in DNMT1g2 mutants (Supplementary Table 1, individuals #5-8), on the other hand, validates the specificity of the antibody, and confirms that mutations in exon 11 indeed result in gene knockout. Furthermore, all mutant ovaries revealed young follicles (Fig. 3C, Supplementary Fig. 5), suggesting that DNMT1 exerts its crucial function after the initiation of oogenesis. However, even though sample sizes are limited, we never observed fully formed oocytes in mutant ovaries, consistent with the absence of mutant G1 eggs. Given that meiosis in *O. biroi* is completed only after oviposition^44^, this suggests that oocyte maturation and meiosis cannot progress without DNMT1 function. The finding that DNMT1 is required for oogenesis in *O. biroi* is consistent with recent findings in the milkweed bug *Oncopeltus fasciatus*^49,52,63^ and the red flour beetle *Tribolium castaneum*^50^, where maternal RNAi knockdown of *DNMT1* leads to sterility or early developmental arrest. Interestingly, this opens the possibility of a DNMT1 function independent of DNA methylation^26,50^.

Based on these findings, we asked whether DNMT1 is present during early oogenesis. Indeed, we observed DNMT1 protein in oocytes within the ovary at multiple stages of development. In very early oocytes, which are several weeks from maturing, DNMT1 is confined to the nucleus (Fig. 3D, top). As oocytes mature, DNMT1 staining also becomes apparent as large puncta at the periphery of the egg that do not appear to be associated with DNA (Fig. 3D, bottom). Together, these experiments show that both DNMT1 protein and mRNA are maternally provisioned into the oocyte at an early stage and support the idea that maternal DNMT1 plays a crucial role in oocyte maturation.

While we cannot rule out the possibility that DNA methylation directly regulates gene expression and alternative splicing in some contexts, our finding that *O. biroi* workers and males develop normally without a functional *DNMT1* gene and with greatly reduced DNA methylation levels shows that, unlike in mammals, this mechanism is not fundamentally required during ant embryogenesis. Instead, the data presented here align with recent evidence that DNMT1 plays a conserved and crucial, yet poorly understood role in insect reproduction^48–52^. Importantly, this role might be independent of DNA methylation^50,63^. DNA methylation-independent functions of DNMT1 have in fact been observed outside of insects. In the African clawed frog *Xenopus laevis*, for example, DNMT1 acts as a direct transcription repressor protein to prevent premature activation of gene expression before the mid-blastula stage^64^. Such DNA methylation-independent functions of DNMT1 could help explain why the enzyme is highly conserved over evolutionary time^4,65^, and why it has been retained even in some insects that do not methylate their DNA^50^. Given its potentially broad relevance for reproductive health across the animal tree of life, future work should aim to better understand the role of DNMT1 during oogenesis.

The fact that DNMT1 loss-of-function mutants in the clonal raider ant are sterile and therefore cannot be propagated led to limitations of the current study. First, with only few mutant individuals available, we had to work with small sample sizes and had to restrict the scope of our experiments. Second, we had to work with G0 individuals, i.e., individuals that were mutagenized at the egg stage and therefore might have been mosaics of mutant alleles, together with wild-type alleles at low frequencies. These limitations could be overcome in the future, e.g. by working with genetically modified strains in which loss of DNMT1 function is inducible. Finally, while we focused our efforts on DNMT1, it remains possible that DNMT3 is involved in regulating caste development via de novo DNA methylation at specific target sites. Studies targeting DNMT3 for loss-of-function could help resolve this question.

## Methods

### Mutagenesis target selection and amplification

The *DNMT1* (LOC105286975) gene locus was located in the published *O. biroi* genome (GenBank assembly accession: GCA_003672135.1)^44^. Two alternate splice variants (X1: XM_011352329, X2: XM_011352333) were identified, and aligned with MAFFT^66^, verifying gRNA sequence presence in both splice variants. Primers were designed to amplify ~200–300 bp fragments surrounding each target cut site (Supplementary Table 4).

### Genotyping and mutant identification

Two *O. biroi* clonal lines, Line A and Line B^43^ were used for this study. All experimental individuals belonged to Line B, and Line A individuals were used as chaperones to rear eggs or larvae. When necessary, genotyping to distinguish between Line A and B was carried out using a restriction enzyme assay of PCR amplicons of the mitochondrial cytochrome oxidase subunit 1 (CO1) gene^45^. Mutants were identified via Sanger sequencing of PCR amplified DNA fragments containing either the DNMT1g1 or DNMT1g2 target locus (Supplementary Table 4) using a previously described protocol^45^. Sanger sequencing was outsourced to the company Psomagen.

### gRNA design and reagent validation

Guide RNAs (gRNAs) were designed using the CRISPR Design Tool (Synthego). For each target, four custom gRNAs were purchased from the company Synthego and tested using an in vitro incubation assay with Cas9 enzyme using PCR amplified and purified DNA from the target region. Reactions were incubated for 4 h at 37 °C, and products were visualized on 2% agarose gels^45^. All gRNAs successfully digested target DNA, and a single gRNA was selected for each gene target for further experiments (Supplementary Table 5).

### CRISPR/Cas9 reagent preparation, mutagenesis and animal rearing

Reagent preparation, egg collection, injection, incubation and animal rearing followed a previously published protocol for this species^45^. The CRISPR/Cas9 injection reagent mix included 100 ng/µl Cas9 (PNABio) and 100 ng/µl of the selected gRNA (Synthego) (Supplementary Table 5).

### DNMT1g1 CRISPR/Cas9

#### DNMT1g1 mutant generation and line propagation

5152 Line B eggs were injected. From those, 76 larvae hatched, and 65 of these G0 larvae were fostered. All G0 adults were pooled in a single colony, which was supplemented with adults from clonal Line A. Eggs from this colony were collected weekly or biweekly and fostered into nests containing 20 Line A chaperones, where they were reared to adulthood. The eclosing callows were individually paint marked^45^ and pooled into six units of 16 ants each. All eggs produced by these units were collected weekly or biweekly and fostered with Line A chaperones to propagate the lines. Mutants resulting from these units were identified via Sanger sequencing of the target region and pooled to generate pure colonies for each unique mutant genotype.

#### DNMT1g1 mutant reproduction and survival

From the first four paint-marked G1 units, eggs were collected biweekly for 2.5 weeks and incubated for 48 h, before they were frozen dry on a glass slide at −80 °C for later DNA extraction and genotyping^45^. All eggs produced after this were removed weekly and fostered with Line A chaperones as described above. All carcasses were removed from the units and frozen dry in a PCR tube at −80 °C until no ants remained. Some carcasses could not be recovered, probably because ants in the colony had dismembered them. For the analysis of reproductive output, only adults (Line A and Line B) and the first 25 eggs (Line A and Line B) sequenced from each unit where mutant adults were observed were included in the analysis (*n* = 3 units). For analysis of survival, DNA was extracted from all frozen adult carcasses for genotyping, and all data from Line A individuals were discarded. Statistical analyses were conducted in GraphPad Prism (v 9.1.1).

### DNMT1g2 CRISPR/Cas9

#### DNMT1g2 mutant generation, reproduction and survival

We injected 2416 Line B eggs, of which 45 hatched. 39 larvae were fostered to yield 9 G0 adults that survived beyond three days after eclosion. One of these adults was a male and was excluded from further analyses. All DNMT1g2 G0 animals from this experiment were placed in a single unit, along with paint-marked Line A chaperone ants to ensure colony stability. All eggs and carcasses were removed from the colony weekly or biweekly and frozen dry at −80 °C on a glass slide or in a PCR tube, respectively. Four carcasses and 24 eggs were collected, and DNA for Sanger sequencing was extracted from whole carcasses and eggs. Additionally, at 61 days, the two remaining G0 ants were sacrificed, ovaries were removed, and DNA was extracted from the remaining tissue. Of the collected eggs, 19 were Line B wild type, four were Line A, and one could not be successfully genotyped and was removed from analyses. No mutant eggs were observed in this experiment. Therefore, unlike for DNMT1g1, we were unable to propagate the mutant lines for DNMT1g2, and all experiments were thus limited to G0 adults. GraphPad Prism (v 9.1.1) was used for statistical analyses.

#### DNMT1g2 mutant generation, morphometrics, immunohistochemistry and whole genome bisulfite sequencing

We injected 3227 eggs in a separate experiment, of which 109 hatched. 92 larvae were fostered to yield four colonies of G0 adults. A single leg was removed from each adult, and DNA was extracted for Sanger sequencing. One of these colonies was excluded from analyses because all six G0s were wild-type. The remaining three colonies generated 18 G0 adults, of which four carried only mutant alleles and 14 carried only wild-type alleles. In parallel, 574 eggs were incubated without injection and reared under identical conditions to yield 17 control adults.

At ~1 week of age, all G0 animals (injected and uninjected) were immobilized in a standardized position (Fig. 2A) under acrylic and imaged using a brightfield microscope (Leica MSV266) for morphometric measurements. A ruler was imaged under identical conditions for accurate calibration. Morphometric measurements of individual body segments were taken using Fiji^67^. All mutants and a subset of wild types were dissected, and their ovaries and brains fixed for immunohistochemistry (next section). After the ovaries and head had been removed, the rest of the body was used for DNA extraction and WGBS (see below).

### Fluorescence in situ hybridization

Tissue was fixed, prepared and processed according to a previously published protocol^68^. Briefly, to prepare the tissue, ovaries were dissected and fixed in cold 4% paraformaldehyde in PBS for at least 2 h at 4 °C, following an EtOH dehydration and incubation overnight. Tissue was rehydrated and incubated in 5% acetic acid for 5 min, followed by another fixation using 2% paraformaldehyde for 1 h at 25 °C. The tissue was washed with 0.5% Tween 20 in PBS and 1% NaBH_4_. Prepared tissue was rinsed before incubation with the pre-hybridization solution for 30 min at 45 °C, and was then incubated with 2pmol of the probes in probe hybridization solution for 48 h. Signal was amplified with 30 pmol snap cooled hairpin solution at room temperature. A set of 30 custom probes were designed for *DNMT1* and purchased from Molecular Instruments Inc. The probes used a B2 HCR amplifier and were labeled with Alexa Fluor 546. Images were captured with an inverted LSM 780 laser scanning confocal microscope (Zeiss). Images were processed in parallel with Fiji^67^ and are shown as maximum projection z-stacks.

### Immunohistochemistry

Tissue was dissected in cold 1xPBS and fixed in 4%PFA for 2 h. Following washes in 1xPBS, samples were blocked using a solution containing 5% normal goat serum in PBSTx for 1 h. The blocking solution was replaced with a primary antibody solution containing 1:100 DNMT1 antibody (Abcam ab188453), and incubated overnight at room temperature. Negative controls without primary antibody were incubated overnight in the blocking solution at room temperature in parallel. All samples were washed and incubated in the secondary solution including 1:500 secondary antibody (Donkey anti-rabbit, AlexaFluor 594, Invitrogen A21207) and 1:500 DAPI for 2 h prior to mounting in DAKO fluorescence mounting medium. Images were captured and processed as described above.

### Whole genome bisulfite sequencing

#### DNA extraction, library construction and sequencing

Genomic DNA was extracted from four mutant and four wild-type replicates of single whole ants (DNMT1g1) or the remaining tissue after brain and ovary dissection (DNMT1g2) using the QIAamp DNA Micro Kit (QIAGEN). While we were able to establish stable lines for DNMT1g1 mutants and thus extract DNA from whole animals, our sample sizes for DNMT1g2 mutants were necessarily limited, and we therefore extracted DNA after brains and ovaries had been removed for other analyses. In each case, all replicates were sequenced to evaluate global methylation changes (Fig. 1C, Supplementary Fig. 1B, 2). Additionally, two of the DNMT1g2 mutant and matched wild-type replicates were selected for more extensive analysis of methylation changes (Fig. 1D–F) using high coverage sequencing (Supplementary Table 2).

MethylC-seq libraries were constructed using the MethylC-seq protocol^69^. Briefly, genomic DNA was sonicated to around 200 bp using a Covaris S-series focused ultrasonicator, and end-repaired with an End-It DNA end-repair kit (Epicentre). The end-repaired DNA was subjected to A-tailing using Klenow 3′–5′ exo − (NEB) and ligated to methylated adapters using T4 DNA ligase (NEB). The ligated DNA was treated with sodium bisulfite reagent using the EZ DNA methylation-Gold kit and amplified using KAPA HiFi uracil + Readymix Polymerase. Sequencing was performed on an Illumina NextSeq500 instrument.

#### Methylome mapping

The MethylC-seq data from the Georgia Genomics & Bioinformatics Core were processed with the “paired-end-pipeline” function in Methylpy^70^. Reads passing quality control filters were aligned to the *O. biroi v5.4* reference genome^71^ using bowtie 2.2.4 (Langmead and Salzberg, 2012), and the uniquely aligned and nonclonal reads were retained. Unmethylated lambda phage DNA was used as a control to calculate the sodium bisulfite conversion rate of unmethylated cytosines. A binomial test was used to determine the methylation status of cytosines with a minimum coverage of five reads.

#### Analysis of DNA methylation

Protein coding genes (*n* = 11,868) and TEs/repeats over 100 bp in size (*n* = 53,158) were used for the plots. For the metaplots in Fig. 1E, genes or TEs/repeats along with 1 kb upstream/downstream regions were divided into 20 bins, and the average methylation level of each bin was displayed. For Fig. 1F, exons, introns, and 1 kb upstream/downstream regions were divided into 20 bins, and the average methylation level of each bin was plotted. For the genome-wide methylation analysis shown in Supplementary Fig. 2, the average percentage of CG methylation in each 50 Kb window was calculated and plotted onto a chromosomal map of the *O. biroi* genome. For the read level analysis in Supplementary Table 3, the output BAM files generated by Methylpy^70^ were used to distinguish wild-type from mutant reads. Each BAM file was visualized with Samtools tview^72^, and mapped mutant and wild-type reads were counted for each sample.

### RNA sequencing

#### RNA extraction, library construction and sequencing

RNA was extracted and eluted in 30 µl RNAse free water from whole ants dry frozen at −80 °C using the RNeasy Mini Kit (QIAGEN Cat. No. 74104). 1 ng of total RNA was used to generate full length cDNA using Clontech’s SMART-Seq v4 Ultra Low Input RNA Kit (Cat # 634888). 1 ng of cDNA was then used to prepare libraries using the Illumina Nextera XT DNA sample preparation kit (Cat # FC-131-1024). Libraries with unique barcodes were pooled at equal molar ratios and sequenced on an Illumina NextSeq 500 sequencer to generate 150 bp paired-end reads, following the manufacturer’s protocol (Cat # 15048776 Rev.E).

#### Analysis of RNA sequencing data

Sequencing reads were trimmed using Trimmomatic^73^ Nextera PE adapters. Trimmed read quality was verified using FastQC, and aligned using STAR^74^ to the *O. biroi v5.4* genome and converted to BAM files with Samtools^72^. BAM files were loaded into Integrative Genomics Viewer^75^ to visualize read alignments at the mutation site and alternative splicing of the *DNMT1* gene. Gene counts were determined with HTseq^76^. Normalization and differential gene expression analysis were carried out in R using DEseq2^77^.

### Statistics and reproducibility

All microscopy images in this paper are representative of multiple examined specimens. The gross anatomy of the *O. biroi* ovary shown in Fig. 3A was observed in 12 samples. The FISH staining shown in Fig. 3B and Supplementary Fig. 4A was consistent across 8 experimental animals and 4 negative controls. The DNMT1 antibody staining shown in Fig. 3C and Supplementary Fig. 4B was consistently observed in 14 experimental animals and 9 negative controls. The DNMT1 antibody staining of DNMT1g1 mutants shown in Fig. 3C was replicated in 6 animals, including both mutant genotypes. DNMT1 antibody staining is shown for 3 different DNMT1g2 mutants in Fig. 3C and Supplementary Fig. 5A, B. The DNMT1 antibody staining pattern in oocytes shown in Fig. 3D was consistent across 13 young and 10 old oocytes from multiple animals. Statistical analyses for other experiments are described in the respective Methods and main text sections, as well as in the figure legends.

### Reporting summary

Further information on research design is available in the Nature Portfolio Reporting Summary linked to this article.

## Supplementary information

Supplementary Information

Reporting Summary

## Data Availability

Whole genome bisulfite sequencing data are available at GenBank/NCBI under accession number GSE182212. RNA-sequencing data are available at GenBank / NCBI under accession number PRJNA780766. All other data are available as Source Data files as part of this publication. Source data are provided with this paper.

## Source data

Source Data

## Code availability

All custom code used for analyses is deposited in the following GitHub repository: https://github.com/schmitzlab/Methylome-of-clonal-ant_Obir-v5.4.git.
