# Supplementary material for: DNMT1 mutant ants develop normally but have disrupted oogenesis
Source: Nat Commun. 2023 Apr 18;14:2201. doi: 10.1038/s41467-023-37945-4 (PMC10113331; doi:10.1038/s41467-023-37945-4)
Supplement: Supplementary file 1 — Supplementary Information [file 41467_2023_37945_MOESM1_ESM.pdf]

Supplementary Information for

DNMT1 mutant ants develop normally, but have disrupted oogenesis

Authors: Iryna Ivasyk*, Leonora Olivos-Cisneros, Stephany Valdés-Rodríguez, Marie Droual, Hosung Jang, Robert J. Schmitz, Daniel J. C. Kronauer*

*Correspondence to: iivasyk@rockefeller.edu; dkronauer@rockefeller.edu

This file contains Supplementary Figures 1-5 and Supplementary Tables 1-5.

Supplementary Figures

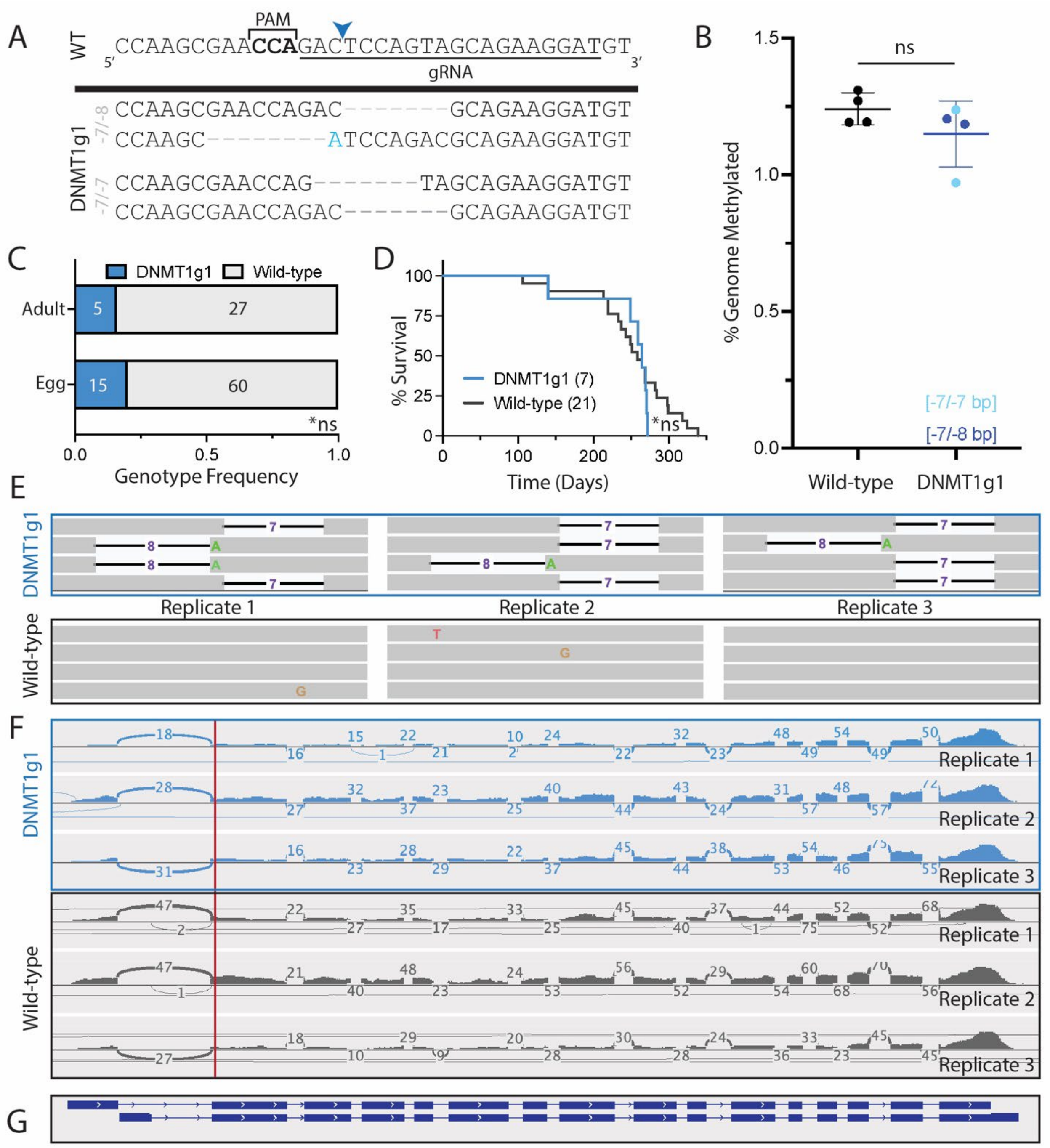

Supplementary Fig. 1. **A frameshift in the second exon of *DNMT1* produces no obvious phenotype. (A)** Top: Wild-type *DNMT1* sequence at DNMT1g1 target site with protospacer adjacent motif (PAM) in bold. Guide-RNA (gRNA) sequence underlined. Blue arrowhead

indicates predicted cut site. Bottom: Two mutant genotypes observed in G1 adults and subsequent generations show small deletions (gray) and base changes (blue) relative to the wild-type sequence around the predicted cut site. **(B)** Genome wide methylation, as determined by WGBS and corrected for bisulfite non-conversion. Genome wide methylation levels in DNMT1g1 mutants are indistinguishable from wild types, ns = no significant difference (two-sided unpaired t-test: p = 0.22; n = 4 animals per condition). Error bars show standard deviation around the mean. Each data point represents DNA extracted from a single whole ant. **(C)** Ratio of DNMT1g1 mutant (of either genotype) to wild-type adults in G1 mixed colonies compared to genotype ratios of eggs collected from the same colonies. Numbers indicate respective sample sizes. No significant difference (*ns) is observed in reproductive output (two-sided Fisher's exact test: p = 0.79). **(D)** DNMT1g1 mutants (G1s) show no significant difference (*ns) in survival compared to wild-types (log-rank test: p = 0.501). Sample sizes indicated in parentheses. **(E)** Four aligned mRNA sequencing reads each from three DNMT1g1 (-7bp/-8bp) mutants (blue) display the expected -8bp and -7bp frameshift deletions compared to reads from three wild-type control animals (black). **(F)** Sashimi plots with read depth of *DNMT1* RNA-seq reads from three DNMT1g1 (-7bp/-8bp) mutants (blue) and three wild types (black). No major alternative splice forms are observed in the DNMT1g1 mutants. Red line indicates mutation site. In the top mutant, a single read skips exon 4. **(G)** Expected mRNA splice variants of wild-type *DNMT1* in *O. biroi* (NCBI Accession: XM_011352333.3, XM_011352329.2). Source data are provided as a Source Data file.

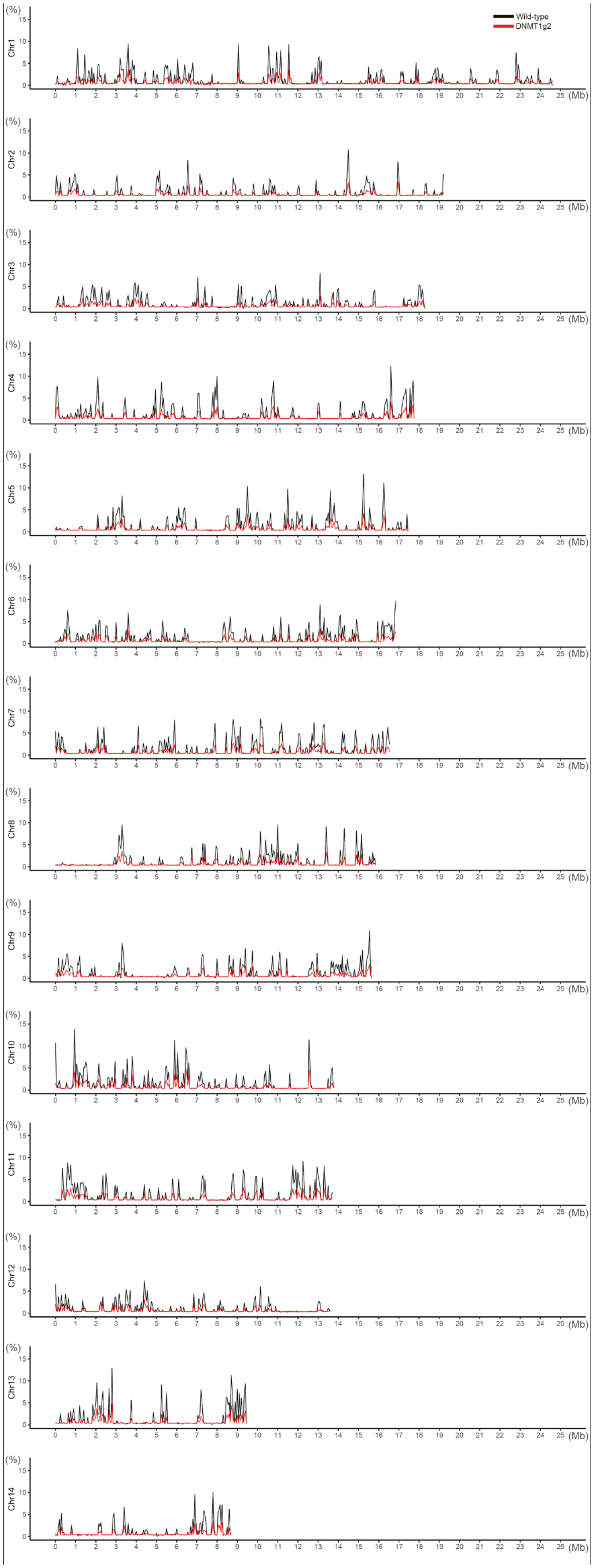
Wild-type
DNMT1g2
(%)
Chr1
Chr2
Chr3
Chr4
Chr5
Chr6
Chr7
Chr8
Chr9
Chr10
Chr11
Chr12
Chr13
Chr14
(Mb)

Supplementary Fig. 2. **Frameshift mutations in the catalytic domain of *DNMT1* result in uniformly decreased methylation levels across the genome.** The average percentage of CG methylation in each 50 Kb window was calculated across the genome and plotted on a chromosome-level assembly of the *O. biroi* genome for wild-type (black) and DNMT1g2 mutants (red). This demonstrates a global decrease of DNA methylation levels in mutants. Source data are provided as a Source Data file.

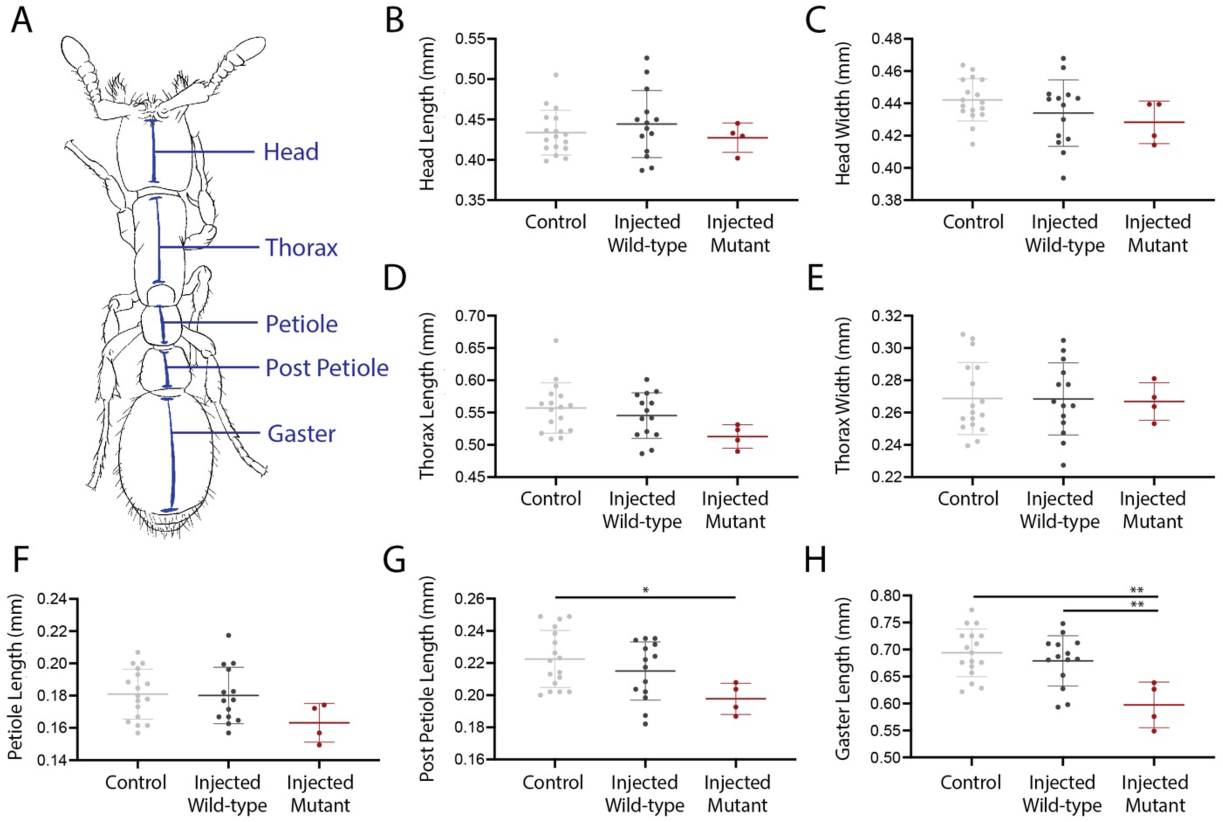

Supplementary Fig. 3. **Morphometric measurements of DNMT1g2 mutants. (A)** Diagram of *O. biroi* anatomy. Morphometric measurements of head, thorax, petiole, post petiole, and gaster comparing G0 mutant (Injected Mutant) to wild-type adults that emerged from injected eggs (Wild type Injected), and wild-type adults from uninjected eggs reared in parallel (Control). **(B-H)** DNMT1g2 mutants and wild types differ in (G) post petiole length (one-way ANOVA p = 0.046) and (H) gaster length (one-way ANOVA p = 0.0022). Horizontal bars indicate significance using Tukey's multiple comparisons tests. No significant differences were observed for the other morphometric measurements (one–way ANOVA). Plots show mean and STD. All analyses are based on n = 4 mutant animals, 17 uninjected wild-type control animals, and 14 wild-type control animals that were injected at the egg stage along with the mutants, but in which no mutations in *DNMT1* were generated. Source data are provided as a Source Data file.

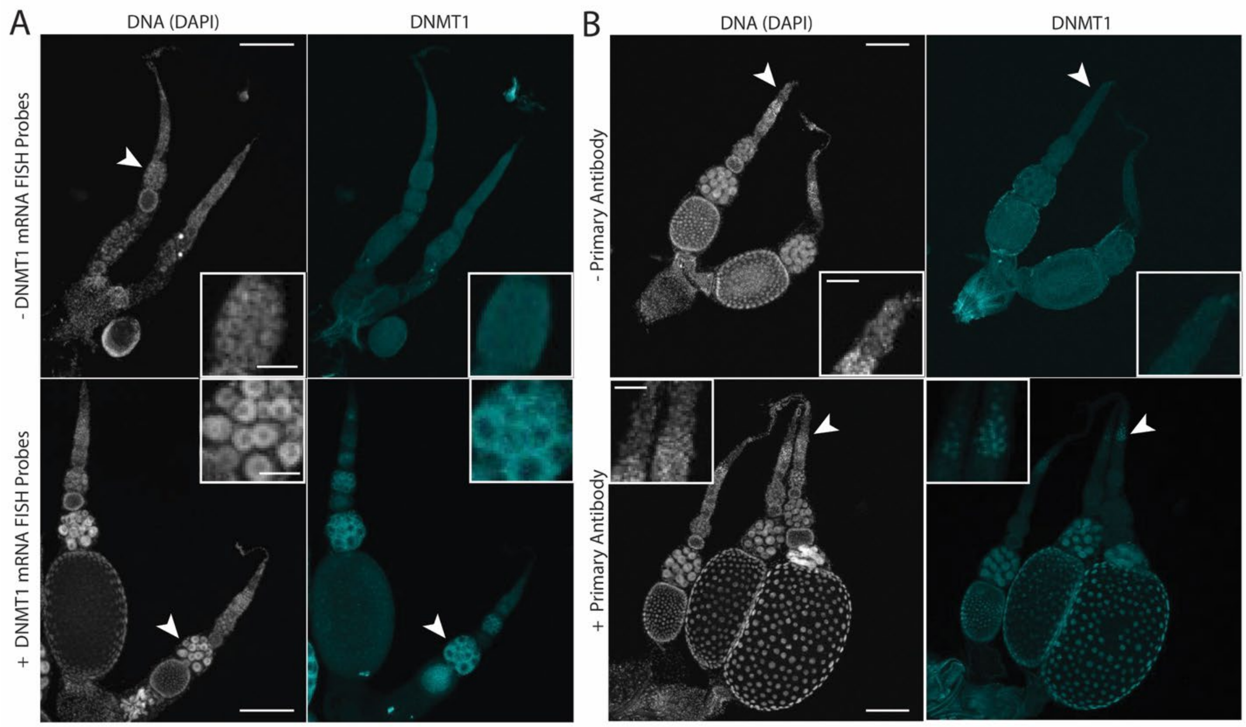

Supplementary Fig. 4. **Negative controls for mRNA fluorescence in situ hybridization and immunohistochemistry. (A) (**Top) *DNMT1* mRNA FISH probes were omitted in the negative control, showing only diffuse autofluorescence in the tissue. (Bottom) Strong signal observed in germarium, nurse cells (arrowheads) and some oocytes with the addition of *DNMT1* mRNA FISH probes (scale bars = 100 µm). Insets show magnified nurse cells (scale bars = 25 µm). Note that the bottom images show the same ovary as Fig. 3B. **(B)** (Top) Tissue incubated in blocking solution instead of primary antibody solution shows diffuse signal from autofluorescence or nonspecific secondary antibody binding. (Bottom) Incubation with primary antibody for DNMT1 protein shows increased signal in oocytes, nurse cells and the germarium (arrowheads). Insets show magnified germarium cells (scale bars = 25 µm). Maximum projection Z stacks shown (scale bars = 100 µm). DNA is stained with DAPI in both (A) and (B).

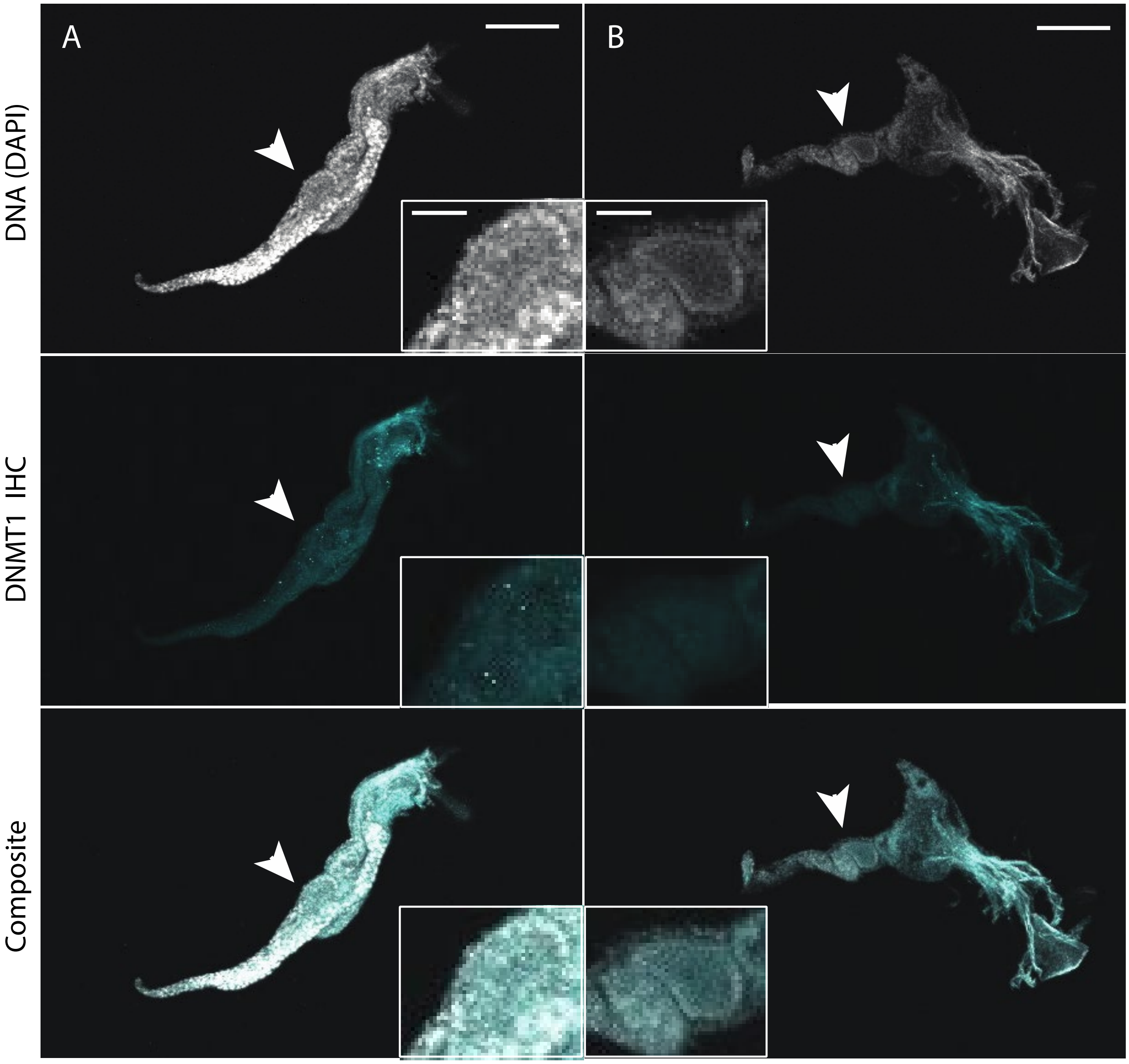

Supplementary Fig. 5. **DNMT1 immunohistochemistry for two additional DNMT1g2 mutant ovaries. (A-B)** DNMT1 shown in cyan, DNA (DAPI) in white. DNMT1 signal is diffuse, likely due to autofluorescence or nonspecific secondary antibody binding, and not elevated in the oocyte, nurse cells or germarium in either mutant. While gross ovarian morphology may deviate from normal, developing oocytes are visible in both cases (arrowheads) (scale bars = 100 μm). Insets show magnified oocytes (scale bars = 25 μm).

Supplementary Tables

#	Alleles Observed (Sanger sequencing)	IHC	Survival [days]	% Genome Methylated
1	[-1bp/+2bp]	N/A	12	N/A
2	[-2bp]	N/A	16	N/A
3	[+2bp/+4bp]	N/A	40	N/A
4	[+1]	N/A	59	N/A
5	[-3bp/-8bp]	N/A	N/A	0.268
6	Unknown	Fig. S6B	N/A	0.211
7	[+2bp/-3bp]	Fig. 3C	N/A	0.268
8	[-2bp/-8bp]	Fig. S6A	N/A	0.288

Supplementary Table 1. **Overview of all female DNMT1g2 mutants with observed alleles and phenotypes**. Mutants were generated in two batches, giving rise to individuals #1-4 and 5-8, respectively. Animals from the first batch (1-4) were used for survival and reproduction experiments. Animals from the second batch (5-8) were used for morphometric measurements before removal of ovaries and brains. The ovaries were used for immunohistochemistry (IHC) and the remaining tissues were used for WGBS. The percent of cytosines methylated across the genome is given after correction for bisulfite non-conversion.

Name	Number of raw reads	Number of aligned reads	Percent of aligned reads	Mapping coverage	Conversion rate (%)
Wild-type-rep1	49,979,978	25,133,592	50.287	13.085	99.643
Wild-type-rep2	56,829,209	29,569,559	52.032	15.668	99.554
DNMT1g2-rep1	50,557,895	26,667,210	52.746	14.720	99.628
DNMT1g2-rep2	71,219,602	28,967,947	40.674	13.718	99.584

Supplementary Table 2. **High coverage whole genome bisulfite sequencing summary statistics.** The number of sequenced reads, aligned reads, and estimated read coverage across the genome. Sodium bisulfite conversion rates were calculated based on unmethylated lambda phage control DNA that was spiked into each sample prior to library preparation. Samples DNMTg2-rep2 and DNMT1g2-rep1 correspond to samples 7 and 8 in Table S1, respectively.

Sample	% wild-type reads	Specimen #
Wild-type-rep1	100% (16/16)	n/a
Wild-type-rep2	100% (40/40)	n/a
DNMT1g2-rep1	0% (0/40)	8
DNMT1g2-rep2	0% (0/36)	7

Supplementary Table 3. **Mutant and wild-type reads in high coverage whole genome bisulfite sequencing data.** All reads from DNMT1g2-rep1 have either a 2-bp or an 8-bp deletion (n = 40), causing frame-shift mutations in the target site. All DNMT1g2-rep2 reads have either a 2-bp insertion (n = 22) or a 3-bp deletion (n = 14). These genotypes are consistent with Sanger sequencing data on leg tissue (Table S1). Reads from wild-type control samples, on the other hand, do not carry any mutations. Specimen # refers to the numbers given in Table S1.

DNMT1g1	FWD	CGTTCGCCTCGGTATTCATC
	REV	AATAAGAGACTGGTGGCACG
DNMT1g2	FWD	ATTAGGGTGATCGCACGGAC
	REV	ATCGCAGTAAGACAAACACGAG

Supplementary Table 4. **PCR primers used to amplify DNMT1g1 and DNMT1g2 mutagenesis target loci.** Forward (FWD) and reverse (REV) primers used to amplify regions in the second exon (DNMT1g1) and the catalytic domain (DNMT1g2) of the *O. biroi DNMT1* gene.

DNMT1g1	1	UUCAACGGAUUCCUCCAUGC
	2	GCUGGCAUGCUUCCGACUAC
	**3**	**AUCCUUCUGCUACUGGAGUC**
	4	GGUUCGCUUGGUACAGUAGG
DNMT1g2	1	CCGCUAAAACCUUGACACGG
	**2**	**GGAGAAGUCGAGCUCCUGUG**
	3	AGCUCGACUUCUCCCUUCUG
	4	UGGACAGAAACUUCCCCAGA

Supplementary Table 5. **Guide RNAs tested for *DNMT1* mutagenesis.** Four gRNAs were tested for each mutagenesis target region. gRNAs selected for generating mutants are shown in bold.
