# Supplementary material for: DNMT1 mutant ants develop normally but have disrupted oogenesis
Source: Nat Commun. 2023 Apr 18;14:2201. doi: 10.1038/s41467-023-37945-4 (PMC10113331; doi:10.1038/s41467-023-37945-4)
Supplement: Supplementary file 2 — Reporting Summary [file 41467_2023_37945_MOESM2_ESM.pdf]

nature portfolio

Corresponding author(s): Daniel Kronauer

Last updated by author(s): Mar 23, 2023

Reporting Summary

Nature Portfolio wishes to improve the reproducibility of the work that we publish. This form provides structure for consistency and transparency in reporting. For further information on Nature Portfolio policies, see our Editorial Policies and the Editorial Policy Checklist.

Statistics

For all statistical analyses, confirm that the following items are present in the figure legend, table legend, main text, or Methods section.

n/a	Confirmed	
☐	☒	The exact sample size (*n*) for each experimental group/condition, given as a discrete number and unit of measurement
☐	☒	A statement on whether measurements were taken from distinct samples or whether the same sample was measured repeatedly
☐	☒	The statistical test(s) used AND whether they are one- or two-sided<br>*Only common tests should be described solely by name; describe more complex techniques in the Methods section.*
☒	☐	A description of all covariates tested
☐	☒	A description of any assumptions or corrections, such as tests of normality and adjustment for multiple comparisons
☐	☒	A full description of the statistical parameters including central tendency (e.g. means) or other basic estimates (e.g. regression coefficient) AND variation (e.g. standard deviation) or associated estimates of uncertainty (e.g. confidence intervals)
☐	☒	For null hypothesis testing, the test statistic (e.g. *F*, *t*, *r*) with confidence intervals, effect sizes, degrees of freedom and *P* value noted<br>*Give P values as exact values whenever suitable.*
☒	☐	For Bayesian analysis, information on the choice of priors and Markov chain Monte Carlo settings
☒	☐	For hierarchical and complex designs, identification of the appropriate level for tests and full reporting of outcomes
☒	☐	Estimates of effect sizes (e.g. Cohen's *d*, Pearson's *r*), indicating how they were calculated

Our web collection on statistics for biologists contains articles on many of the points above.

Software and code

Policy information about availability of computer code

Data collection

no software was used for data collection

Data analysis

Commercial software was used for data analysis including:
GraphPad Prism (9.1.1)
MAFFT (V7.486)
Fiji (ImageJ v 2.1.0/1.53c, build 5f23140693)
Methylpy
bowtie (v 2.2.4)
Samtools tview (v 1.9)
Trimmomatic (v 0.36)
FastQC (v 0.11.5)
STAR (v 2.5.0a)
Samtools (v 1.8)
Integrative Genomics Viewer (v 2.10.0)
HTseq (v 0.9.1)
R using DEseq2 (v 1.3.0)
CRISPR design tool (Synthego) v 1

The MethylC-seq data was processed using Methylpy (v1.4.2), and the output allc files were used for calculating average weighted methylation (Schultz et al. 2012) for different genomic features (custom code). To analyze genic DNA methylation patterns, each primary transcript was extracted from the annotation data (custom code), which was used for calculating average weighted methylation levels of the

genes (custom code). To analyze the mapped reads for each genotype, Samtools (v1.9) was used to visualize the output BAM file generated by Methylpy. All the custom codes used in this analysis are deposited in the following GitHub repository (https://github.com/schmitzlab/Methylome-of-clonal-ant_Obir-v5.4.git).

Schultz MD, Schmitz RJ, Ecker JR (2012) 'Leveling' the playing field for analyses of single-base resolution DNA methylomes. Trends Genet. 28: 583-585. https://pubmed.ncbi.nlm.nih.gov/23131467/

For manuscripts utilizing custom algorithms or software that are central to the research but not yet described in published literature, software must be made available to editors and reviewers. We strongly encourage code deposition in a community repository (e.g. GitHub). See the Nature Portfolio guidelines for submitting code & software for further information.

Data

Policy information about availability of data

All manuscripts must include a data availability statement. This statement should provide the following information, where applicable:

- Accession codes, unique identifiers, or web links for publicly available datasets
- A description of any restrictions on data availability
- For clinical datasets or third party data, please ensure that the statement adheres to our policy

Whole genome bisulfite sequencing data are available at GenBank / NCBI under accession number GSE182212. RNA-sequencing data are available at GenBank / NCBI under accession number PRJNA780766.

Human research participants

Policy information about studies involving human research participants and Sex and Gender in Research.

Reporting on sex and gender	n/a
Population characteristics	n/a
Recruitment	n/a
Ethics oversight	n/a

Note that full information on the approval of the study protocol must also be provided in the manuscript.

Field-specific reporting

Please select the one below that is the best fit for your research. If you are not sure, read the appropriate sections before making your selection.

☒ Life sciences ☐ Behavioural & social sciences ☐ Ecological, evolutionary & environmental sciences

For a reference copy of the document with all sections, see nature.com/documents/nr-reporting-summary-flat.pdf

Life sciences study design

All studies must disclose on these points even when the disclosure is negative.

Sample size

Sample sizes could not be determined a priori and were limited by the number of mutant ants produced in each of two batches. Given the technical challenges associated with producing mutant ants and the fact that mutants were sterile and could not be propagated, the breadth of possible analyses was limited in this study. However, sample sizes were sufficient to obtain several statistically significant results. Sample sizes for each experiment are detailed in the manuscript text and a supplementary table.

Data exclusions

For the experiment on reproductive output of DNMT1g1 mutants, eggs were collected from four colonies. However, data from one colony was excluded because all sequenced adults were wild-type (i.e., no direct comparison to mutants was possible). For the experiment on longevity, data from individuals that could not be genotyped post mortem were excluded.

For DNMT1g2 mutants, one mutant animal had both wild-type and mutant alleles (a clear mosaic) and one was male; both individuals were excluded from analysis. We were unable to obtain genotypes for two of the G0 animals, which were therefore excluded from all analyses. For the experiment on reproductive output, one egg could not be genotyped and was excluded from analysis. For the immunohistochemistry experiment, one mutant was excluded because the ovaries were lost during the tissue fixation step.

All data from Clonal Line A individuals, which served as chaperones for the experimental ants from Clonal Line B, were excluded from all analysis.

Replication

It was not possible to repeat entire experiments, due to the technical challenges of producing mutant ants. Within each experiment, results were consistent across replicate samples, and all attempts at replication were successful. All microscopy images are representative of multiple examined specimens. A "Statistics and Reproducibility" statement is included in the paper.

March 2021

Randomization	For the experiments described here, animals were not allocated to treatment groups in a controlled manner. Instead, all mutant individuals generated in a given batch were compared to matched control animals from the same batch in which mutagenesis had not been successful.
Blinding	While collecting data on reproduction and longevity, the animals were not genotyped until after the conclusion of the experiment, i.e., data were collected blindly with respect to genotype, and investigators were therefore blinded to group allocation during data collection and entry. For body length and size measurements, wild-type and mutant animals were numbered, photographed and measured blindly with respect to genotype.

Reporting for specific materials, systems and methods

We require information from authors about some types of materials, experimental systems and methods used in many studies. Here, indicate whether each material, system or method listed is relevant to your study. If you are not sure if a list item applies to your research, read the appropriate section before selecting a response.

Materials & experimental systems

n/a	Involved in the study
☐	☒ Antibodies
☒	☐ Eukaryotic cell lines
☒	☐ Palaeontology and archaeology
☐	☒ Animals and other organisms
☒	☐ Clinical data
☒	☐ Dual use research of concern

Methods

n/a	Involved in the study
☒	☐ ChIP-seq
☒	☐ Flow cytometry
☒	☐ MRI-based neuroimaging

Antibodies

Antibodies used	Recombinant Anti-Dnmt1 antibody (Abcam ab188453)<br>Donkey anti-rabbit, AlexaFluor 594 (Invitrogen A21207)
Validation	Supplementary Fig. 4 and Supplementary Fig. 5 demonstrate our validation for the antibody. In wild-type animals shown in Supplementary Fig. 4, the antibody marks nurse cells and cells in the germarium. This staining disappears when the primary antibody is removed. Supplementary Fig. 5 shows that animals with mutations in DNMT1 do not have antibody staining in the nurse cells or germarium, demonstrating specificity of the antibody.

Animals and other research organisms

Policy information about studies involving animals; ARRIVE guidelines recommended for reporting animal research, and Sex and Gender in Research

Laboratory animals	Clonal raider ants, Ooceraea biroi. Clonal line B. Age of animals: For longevity experiments, ant were aged for up to ca. 1 year. For experiments on reproduction, eggs were collected from ants that were between ca. 2 weeks and ca. 1.5 months old. Antibody and FISH staining on wild-type ants was done with mature animals (older than ca. 2 weeks) of unspecified age. DNMT1g2 mutants were ca. 1-2 weeks old when used for WGBS, morphometrics, and immunohistochemistry. For experiments on reproduction and survival, DNMT1g2 mutant animals were between ca. 1 week and ca. 2 months old. RNA-seq and WGBS on DNMT1g1 mutants was conducted on ca. 1 month old animals.
Wild animals	No wild animals were used in this study.
Reporting on sex	The study was limited to females because all the ants in a colony are female. Males in ants occur only sporadically and do not participate in regular colony life. Additionally, our study species is asexual, and males are only produced as very rare "accidents".
Field-collected samples	No field collected samples were used in this study.
Ethics oversight	No ethical guidance or approval was required because this research deals with invertebrates (insects) that are not regulated in this respect.

Note that full information on the approval of the study protocol must also be provided in the manuscript.
